# Vascular Ultrasound for In Vivo Assessment of Arterial Pathologies in a Murine Model of Atherosclerosis and Aortic Aneurysm

**DOI:** 10.3390/ijms242015261

**Published:** 2023-10-17

**Authors:** Alexander Hof, Henning Guthoff, Maysam Ahdab, Max Landerer, Jasper Schäkel, Jana Niehues, Maximilian Schorscher, Oscar Zimmermann, Holger Winkels, Philipp von Stein, Simon Geißen, Stephan Baldus, Matti Adam, Martin Mollenhauer, Dennis Mehrkens

**Affiliations:** 1 Department for Experimental Cardiology, Faculty of Medicine, University of Cologne, Clinic III for Internal Medicine, University Hospital Cologne, 50937 Cologne, Germany; alexander.hof@uk-koeln.de (A.H.); myasam.ahdab74@gmail.com (M.A.); maxlanderer2@gmail.com (M.L.); jschaeke@smail.uni-koeln.de (J.S.); jana.niehues@uk-koeln.de (J.N.); maximilian.schorscher@uk-koeln.de (M.S.); oscar.zimmermann@uk-koeln.de (O.Z.); holger.winkels@uk-koeln.de (H.W.); philipp.von-stein@uk-koeln.de (P.v.S.); simon.geissen@uk-koeln.de (S.G.); stephan.baldus@uk-koeln.de (S.B.); matti.adam@uk-koeln.de (M.A.); martin.mollenhauer@uk-koeln.de (M.M.); 2 Center for Molecular Medicine Cologne (CMMC), Faculty of Medicine and Faculty of Mathematics and Natural Sciences, University of Cologne, 50937 Cologne, Germany

**Keywords:** vascular ultrasound, abdominal aortic aneurysm, atherosclerosis, collagen quantification

## Abstract

Vascular diseases like atherosclerosis and abdominal aortic aneurysm (AAA) are common pathologies in the western world, promoting various potentially fatal conditions. Here, we evaluate high-resolution (HR) ultrasound in mouse models of atherosclerosis and AAA as a useful tool for noninvasive monitoring of early vascular changes in vivo. We used Apolipoprotein E-deficient (*ApoE*^−/−^) mice as an atherosclerosis model and induced AAA development by the implementation of Angiotensin II-releasing osmotic minipumps. HR ultrasound of the carotid artery or the abdominal aorta was performed to monitor vascular remodeling in vivo. Images were analyzed by speckle tracking algorithms and correlated to histological analyses and subsequent automated collagen quantification. Consistent changes were observed via ultrasound in both models: Global radial strain (GRS) was notably reduced in the AAA model (23.8 ± 2.8% vs. 12.5 ± 2.5%, *p* = 0.01) and in the atherosclerotic mice (20.6 ± 1.3% vs. 15.8 ± 0.9%, *p* = 0.02). In mice with AAA, vessel distensibility was significantly reduced, whereas intima–media thickness was increased in atherosclerotic mice. The area and collagen content of the tunica media were increased in diseased arteries of both models as measured by automated image analysis of Picrosirius Red-stained aortic sections. Correlation analysis revealed a strong correlation of multiple parameters, predicting early vascular damage in HR ultrasound and histological examinations. In conclusion, our findings underscore the potential of HR ultrasound in effectively tracing early alterations in arterial wall properties in murine models of atherosclerosis and AAA.

## 1. Introduction

Atherosclerosis and aortic aneurysms are severe pathologies of the vasculature that both alter the structural composition of the arterial wall and limit prognosis by propagating potentially fatal conditions such as myocardial infarction or aortic dissection [1,2,3]. Despite extensive research over recent decades, the underlying pathomechanisms remain incompletely understood. Over recent decades, extensive research has been dedicated to understanding the pathomechanisms of cardiovascular diseases, yet they remain not fully elucidated. Numerous animal models have been developed to investigate the mechanisms behind the onset of such diseases. Preclinical mouse models, particularly those deficient in APOE and LDLR, have played a pivotal role in exploring atherosclerosis and aneurysm development. In addition, different strategies, including enzymatic digestion, application of calcium chloride or angiotensin II, and xenografting, have been evaluated for aneurysm formation in mice. They have been invaluable in validating potential treatments, including lipid-lowering and anti-inflammatory drugs. These models strive to emulate the human condition, hallmarked by chronic inflammation, endothelial dysfunction, and lipid accumulation, which collectively contribute to arterial plaque formation and aneurysm progression. While the measurement of aneurysm diameter is traditionally the primary metric to assess aneurysm progression, emerging imaging modalities and parameters are broadening our understanding and offering deeper insights into aneurysm behavior [4,5]. Though histological assessment of arterial disease phenotypes is a well-established method, it only allows for the evaluation of the vasculature under static conditions at a specific time point. Additionally, the intricacies of sample preparation can introduce anomalies, complicating the interpretation of results. In addition, hemodynamic factors like blood pressure, blood flow or viscosity, and fluctuating concentrations of various endocrine mediators may influence vascular function in vivo but may not be captured by histological examination [6,7]. Hence, in vivo analysis of the vasculature is indispensable.

Ultrasound has emerged as a diagnostic option frequently employed for in vivo assessment of vascular function in animal models. Its primary advantage over postmortem analysis is the ability to determine multiple vascular parameters that reflect the influences mentioned above in vivo. This facilitates a longitudinal approach, useful in contexts such as drug testing or evaluating dose tolerance and efficacy. In addition to vascular wall morphology, e.g., by measuring intima–media thickness (IMT) as a correlate of atherosclerotic wall changes, physiological parameters like pulse wave velocity (PWV) or arterial elasticity can be assessed [8]. In recent years, the technical features of ultrasound devices have drastically improved, allowing accurate vascular measurements in small animal models. The Vevo3100 system was specifically designed for preclinical studies in animal models, providing an improved image quality by automated respiratory gating and HR imaging. By using ultra-high frequencies of maximal 70 MHz, a spatial resolution of up to 30 µm can be achieved, thus enabling the investigator to visualize and trace considerably small structures within the vessel wall [9].

Here, we aim to evaluate and validate the sonographic assessment of atherosclerosis and aortic aneurysm development in mice by the Vevo3100 system. The early functional vessel alterations assessed by ultrasound analysis are compared to and correlated with histological analyses and observer-independent, automated collagen quantification to confirm morphological changes in the arterial wall.

## 2. Results

### 2.1. Vascular Ultrasound Analysis Traces Arterial Wall Changes in Abdominal Aortic Aneurysm

**Figure 1:**
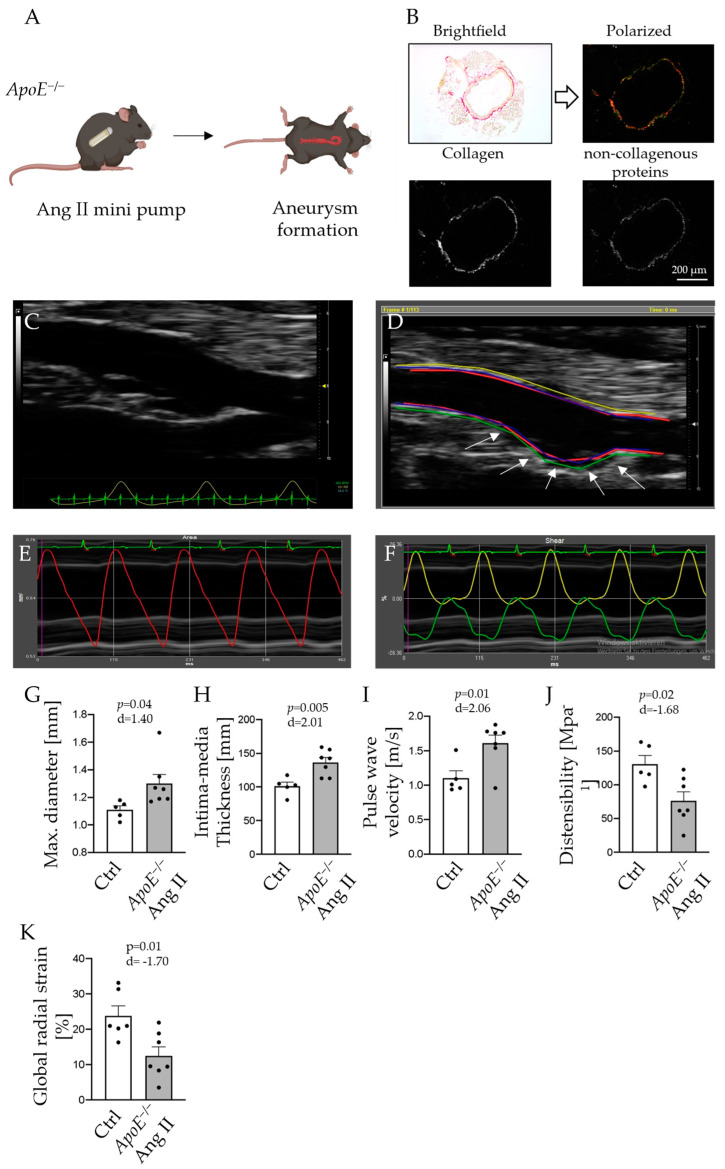
*ApoE*^−/−^ mice were fed a high-cholesterol Western diet for 12 weeks to induce atherosclerosis (**A**). Picrosirius Red-stained aortas were visualized in brightfield and polarized light for collagen quantification (**B**). Binarization by splitting red and green channels was utilized to separate collagen (red) and non-collagen proteins (green). White pixels in the binarized images were counted to determine total collagen within the samples. Paravertebral view in B-Mode of the abdominal aortas in control (**C**) and *ApoE*^−/−^ mice after 28 days of Ang II infusion (**D**); arrows indicate abdominal aortic aneurysm). Longitudinal images of the aorta were acquired. Borders of the vessel wall were defined in VevoVasc 1.2 software, and movement of lumen-intima border (**E**), ventral (green), and dorsal (yellow) outer vessel wall were traced by speckle tracking (**F**). Maximum diameter (**G**), intima–media thickness (**H**), pulse-wave velocity (**I**), wall distensibility (**J**), and global radial strain (**K**) of the carotid arteries are presented for Ang II-infused *ApoE*^−/−^ mice and control animals; *n* = 5–7. Scale bar: 200 µm.

After AAA induction by angiotensin II (Ang II) infusion for 28 days (Figure 1A), the abdominal aorta was assessed by automated collagen quantification and HR vascular ultrasound in Apolipoprotein E deficient (*ApoE*^−/−^) mice (Figure 1B,C). Morphological and functional analyses of the arterial wall were performed with VevoVasc 1.2 software (representative recordings are shown in Figure 1D–F). Ang II-infused *ApoE*^−/−^ mice showed significantly larger maximum diameters compared to controls, with distinguishable aneurysmal segments of the abdominal aorta (1.1 ± 0.03 mm vs. 1.3 ± 0.07 mm, *p* = 0.04; Figure 1G). IMT was enlarged in AAA compared to controls (101.2 ± 5.9 µm vs. 136.7 ± 7.1 µm, *p* = 0.005; Figure 1H). Moreover, PWV was increased in the aneurysmal aorta, indicating higher arterial wall stiffness compared to controls (1.10 ± 0.11 m/s vs. 1.61 ± 0.12 m/s, *p* = 0.01; Figure 1I). Distensibility (130.6 ± 12.8 MPa^−1^ vs. 76.2 ± 13.1 MPa^−1^, *p* = 0.02; Figure 1J) and global radial strain (GRS; 23.8 ± 2.8% vs. 12.5 ± 2.5%, *p* = 0.01; Figure 1K) were decreased, indicating reduced aortic elasticity.

### 2.2. Morphologic and Functional Alterations of the Carotid Artery in Atherosclerosis Manifest in Vascular Ultrasound Measurements

**Figure 2:**
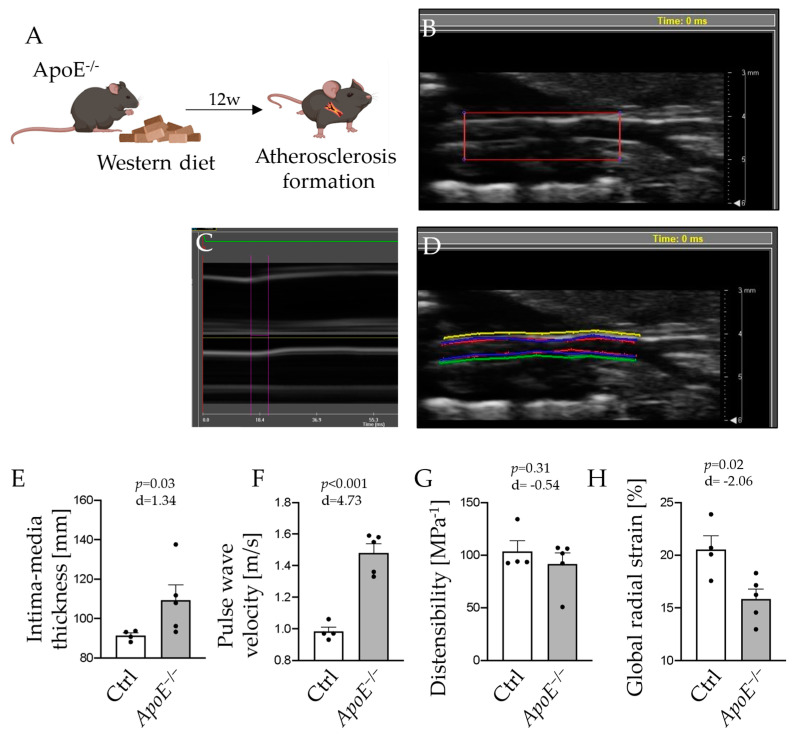
Angiotensin II (Ang II) was infused via osmotic minipumps for 28 days to provoke formation of abdominal aortic aneurysm in *ApoE*^−/−^ mice (**A**). Segments of the carotid artery were examined from its origin to carotid bifurcation ((**B**) longitudinal acquisition of carotid artery). Pulse wave velocity (PWV) was examined in M-mode, measuring time differences in vessel excursion in different segments of the carotid artery (**C**). Vessel demarcation was performed at the luminal and adventitial sites. Distensibility and strain analysis were performed by speckle tracking using the VevoVasc software (**D**). Results for intima–media thickness (**E**), PWV (**F**), wall distensibility (**G**), and global radial strain (**H**) of the carotid arteries are presented for Western diet-fed *ApoE*^−/−^ mice and control animals; *n* = 4–5.

To assess properties of the arterial wall in *ApoE*^−/−^ mice after 12 weeks of Western diet feeding (Figure 2A), ultrasound analysis of the carotid artery was performed (representative recordings are shown in Figure 2B–D). IMT was significantly increased in atherosclerotic *ApoE*^−/−^ mice compared to controls (91.4 ± 1.3 µm vs. 109.3 ± 7.9 µm, *p* = 0.03; Figure 2E). In addition, PWV was significantly accelerated in those animals (0.98 ± 0.03 m/s vs. 1.48 ± 0.06 m/s, *p* < 0.001; Figure 2F), indicating arterial stiffening.

Likewise, parameters of arterial wall elasticity were reduced in *ApoE*^−/−^ mice fed a Western diet. The distensibility of the arterial wall was numerically lower (103.5 ± 10.2 MPa^−1^ vs. 91.6 ± 10.6 MPa^−1^, *p* = 0.31; Figure 2G) and GRS was significantly diminished in atherosclerotic mice (20.6 ± 1.3% vs. 15.8 ± 0.9%, *p* = 0.02; Figure 2H).

### 2.3. Aortic High Resolution Ultrasound Analysis Is Consistent with Histological Postmortem Evaluation of Vascular Disease

**Figure 3:**
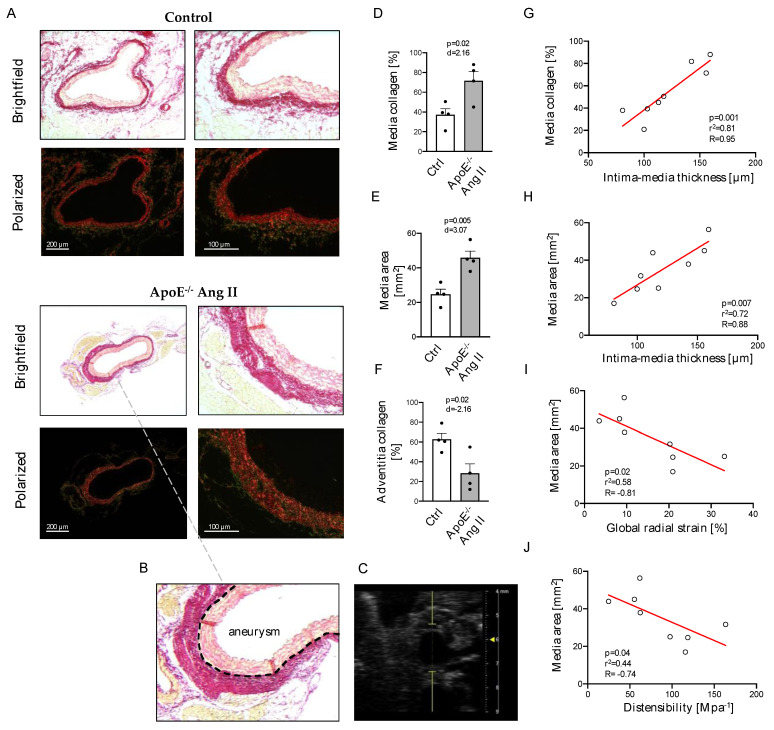
Representative images of control and AAA mice in brightfield and polarized light (**A**) with histological (**B**) and sonographic (**C**) cross-section of AAA region. Collagen content (**D**) and area (**E**) of the tunica media and collagen content of the tunica adventitia (**F**) as assessed by automated analysis of Picrosirius Red-stained cross-sections. Correlation of media collagen content with intima–media thickness (**G**) and of media area with intima–media thickness (**H**), global radial strain (**I**), and distensibility (**J**) as assessed by high-resolution ultrasound. Scale bars: 200 µm (**left panel**) and 100 µm (**right panel**); *n* = 4.

In the AAA model, HR vascular ultrasound analysis was compared to collagen content in Ang II treated ApoE^−/−^ animals and control mice (Figure 3A–C). Collagen content was markedly increased in the media of AAA mice (37.2 ± 6.1% vs. 71.6 ± 9.5%, *p* = 0.02; Figure 3D). The area of the tunica media was significantly enlarged in the AAA tissue of *ApoE*^−/−^ mice in comparison to controls (12.3 ± 3.3 mm^2^ vs. 23.3 ± 6.3 mm^2^, *p* = 0.005; Figure 3E). In addition, the collagen content of the adventitia was significantly lower in the aortic wall of AAA compared to controls (62.8 ± 6.1% vs. 28.4 ± 9.5%, *p* = 0.02; Figure 3F).

In a Spearman correlation analysis, collagen content, as well as the area of the tunica media, correlated significantly with IMT measured by vascular ultrasound (Figure 3G,H). Likewise, the media area in aneurysmal diseased aortic segments significantly correlated with HR ultrasound-obtained markers of arterial stiffening, in particular global radial strain and wall distensibility (Figure 3I,J). Further correlation analysis of sonographic and histological parameters are listed in the Supplement (Figure S1).

**Figure 4:**
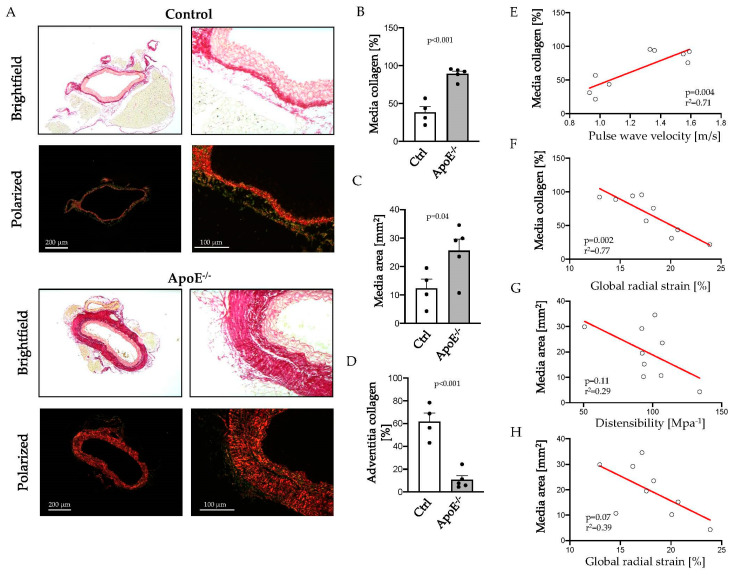
Aortic sections of *ApoE*^−/−^ mice fed a Western diet and controls were stained with Picrosirius Red, and collagen content was visualized in polarized light (**A**). Representative images show collagen content (**B**) and area (**C**) of the tunica media and collagen content of the tunica adventitia (**D**) as assessed by automated image analysis. Correlation of media collagen content with pulse wave velocity (**E**) and global radial strain (**F**) and of media area with distensibility (**G**) or global radial strain (**H**) as assessed by high-resolution ultrasound. Scale bars: 200 µm (**left panel**) and 100 µm (**right panel**); *n* = 4–5.

In the atherosclerosis model, sections of the thoracic aorta were analyzed for collagen content by Picrosirius Red (PSR) staining (Figure 4A). Media collagen content doubled in the thoracic aortas of *ApoE*^−/−^ mice, compared to control animals (38.4 ± 7.7% vs. 89.3 ± 3.6%, *p* < 0.001; Figure 4B), showing significant vascular remodeling in atherosclerosis. Vascular ultrasound demonstrated IMT thickening in Western diet-fed *ApoE*^−/−^ mice with a larger media area (12.3 ± 3.3 vs. 25.6 ± 9.2 mm^2^, *p* = 0.04; Figure 4C) compared to controls. Adventitia displayed a significant reduction in collagen fibers in *ApoE*^−/−^ mice after 12 weeks of Western diet (Figure 4D).

Spearman correlation analysis revealed a significant correlation of media collagen content with PWV and GRS (Figure 4E,F). Arterial wall distensibility and GRS tended to be associated with the area of tunica media, as assessed histologically, but failed to reach statistical significance (Figure 4G,H). Further correlation analysis of sonographic and histological parameters are listed in the Supplement (Figure S2).

## 3. Discussion

In this study, we show that HR vascular ultrasound is a powerful tool for morphological and functional assessment of different vascular pathologies in small animal models, providing information on physiological parameters of arterial function beyond histological or ex vivo examinations. For the first time, we demonstrate multiple parameters of HR ultrasound to significantly correlate with automated and observer-independent histological analysis of aortic collagen content and morphology.

Here, we investigated two mouse models of atherosclerosis and AAA in *ApoE*^−/−^ mice. After 12 weeks of a Western diet, the IMT of the carotid artery and the area of the tunica media in the thoracic aorta were increased in *ApoE*^−/−^ mice, indicating atherosclerotic wall changes [10]. Automated collagen quantification revealed greater collagen content in the media of atherosclerotic mice. These structural changes have previously been described as characteristic features in atherosclerosis contributing to arterial stenosis [11]. Hence, excessive collagen accumulation might directly link to increased PWV as a surrogate for arterial stiffness and markers of reduced arterial wall elasticity, such as global radial strain. Human studies revealed arterial strain values to be reliable predictors of early atherosclerotic wall changes, which significantly correlated with IMT [12]. In our study, simple regression analysis confirmed a strong correlation of different functional parameters and morphological characteristics as assessed by automated histological examination, especially for media collagen content and PWV as well as GRS.

Similarly, strain analysis of the aortic wall by speckle tracking has been proposed for the characterization and risk stratification of AAA in humans. Derwich et al., showed drastically reduced circumferential wall strain in AAA patients compared to young, healthy individuals [13]. With HR vascular ultrasound, we observed a reduction of radial strain and aortic wall distensibility in *ApoE*^−/−^ mice with AAA after Ang II infusion. In line with previous findings, we further demonstrate increased PWV in AAA mice, suggesting a reduction in elastic properties and stiffening of the aneurysmal aorta, which correlate with ultrastructural changes of the extracellular matrix [14,15]. Aortic wall distensibility may predict the risk of infrarenal AAA rupture in later stages of the disease, with higher distensibility measures correlating with a greater risk of rupture, reflecting the observed physiological relevance of increased collagen accumulation in AAA [16,17]. Since AAA rupture occurs in various animal models and aortic dissection or fatal rupture can be triggered, for instance, by Ang II infusion in osteoprotegerin-deficient mice [18], HR ultrasound might be useful to detect early changes in the aortic wall and predict aortic rupture or dissection. In this context, recent data from a small patient cohort study suggest that parameters of aortic wall stress and morphology predict rupture risk even more accurately than the AAA diameter [19]. Thus, HR vascular ultrasound may facilitate similar risk calculations in various animal models of AAA based on the assessment of biomechanical parameters [20,21]. A few studies have compared the parameters of vascular ultrasounds of AAA to the histological assessment of the diseased vessel and delivered heterogeneous results, e.g., on collagen deposition and degradation of the medial layer [14,20,22]. Hence, combining HR vascular ultrasound with automated collagen quantification provides a novel approach to accurately characterize the AAA phenotype. We found PWV and media thickness to be increased in Ang II-infused *ApoE*^−/−^ mice with AAA. Similar observations were made by Busch et al., describing elevated intima–media thickness in surgical, elastase-induced AAA mouse models, but also in human aortas of AAA patients [23]. Likewise, PWV is increased in the aortas of AAA patients [24]. In animals with AAA, we observed a reduced collagen content of the adventitia compared to controls. In line with these results, sparse and disarrayed collagen fibers have been identified by electron microscopy in the adventitia of human AAA [25].

This study has some limitations. First, the causes of AAA development are heterogeneous and include genetic predisposition and infectious and inflammatory conditions. Hemodynamic characteristics and changes in wall stress or morphology of the aneurysmal aorta might differ in those diseases from the *ApoE*^−/−^ Ang II mouse model utilized, in which AAA is induced by increased blood pressure levels, hyperlipidemia, and atherosclerotic predisposition. Likewise, changes in collagen content and aortic stiffness might be altered in enzymatically induced AAA models and also in other models of atherosclerosis [26]. Finally, findings from animal models regarding hemodynamics and morphologic characteristics of the diseased aorta cannot be fully extrapolated to the conditions in humans, and the study is restricted due to the limited sample size, requiring further investigation. However, here, we present a valid method for in vivo assessment of murine vascular pathologies that is consistent with established disease markers assessed by observer-independent, automated histological analysis.

## 4. Materials and Methods

### 4.1. Animal Models

Eight-week-old male *ApoE*^−/−^ mice on a C57Bl6/J background [27] were fed a Western diet (sniff, 1.25% cholesterol diet, E-15723-347, Soest, Germany) for 12 weeks to induce atherosclerosis (Figure 1A(I)). For the induction of abdominal aortic aneurysm (AAA), *ApoE*^−/−^ mice were treated with Ang II for 28 days via osmotic minipumps as outlined below in detail. C57Bl6/J wildtype (WT) animals served as controls. All animal studies were approved by the local Animal Care and Use Committees (Ministry for Environment, Agriculture, Conservation and Consumer Protection of the State of North Rhine-Westphalia: State Agency for Nature, Environment and Consumer Protection (LANUV), NRW, Germany; licenses 81-02.04.2018.A216 and 81-02.04.2020.A249) and were in accordance to the guidelines from Directive 2010/63/EU of the European Parliament on the protection of animals used for scientific purposes.

### 4.2. Implantation of Osmotic Minipumps

For induction of AAA formation, osmotic minipumps were implanted in eight-week-old ApoE^−/−^ mice (Figure 1A(II)). Alzet osmotic minipumps (model 2004; ALZA Scientific Products, Cupertino, CA, USA) were filled with Ang II (Sigma Chemical Co., St. Louis, MO, USA) dissolved in 0.9% saline to deliver 1000 ng/min/kg of Ang II subcutaneously for 28 days, or with 0.9% saline solution only for controls. Mice were anesthetized by isoflurane inhalation (Isofluran-Piramal^®^, Piramal Critical Care, Voorschoten, The Netherlands; 5% vol/vol for induction and 2% vol/vol for maintenance of anesthesia) and analgetic treatment via subcutaneous injection of buprenorphine (TEMGESIC^®^, Indivior Europe Limited, Dublin, Ireland; 0.1 mg per kg body weight) prior to implantation. Subsequently, minipumps were implanted subcutaneously into the left flank of mice.

### 4.3. Ultrasound Analysis

After 12 weeks of Western diet feeding, mice were anesthetized with 5% isoflurane and kept under anesthesia with 2% isoflurane. Ultrasound analysis was performed under continuous monitoring of vital signs. Sequences of the carotid artery were acquired, reaching from the aorta to the carotid bifurcation in B-Mode, M-Mode, and ECG-triggered kilohertz visualization (EKV) using a Vevo3100 device and an MX550S transducer (VisualSonics, Fujifilm, Tokyo, Japan). For examination of the abdominal aorta after 28 days of Ang II infusion, mice were fixed in a prone position, and sequences of the longitudinal axis of the abdominal aorta were acquired from paravertebral in B-Mode, M-Mode, and EKV. B-Mode images of the carotid artery and the abdominal aorta were examined using VevoVasc 1.2 software (VisualSonics, Fujifilm, Tokyo, Japan). Respiratory gating was performed to minimize artifacts. Subsequently, the borders of the vessel wall and intima–media layer were defined. Advanced speckle tracking analysis of moving image sequences allowed evaluation of elastic properties, particularly wall distensibility and GRS, of the vessel wall. Further, PWV as a measure of arterial stiffness [28] was defined by analyzing ECG-triggered excursion of the arterial wall at two distinct locations of a pre-defined distance in longitudinal acquisitions. Examiners were blinded to genotypes and treatments during acquisition and analysis.

### 4.4. Tissue Preparation

The abdominal cavity was opened under deep anesthesia and analgesia as described above, and animals were euthanized by cardiac exsanguination from the left ventricular cavity and consecutive perfusion with ice-cold phosphate-buffered saline (PBS). Subsequently, the aorta was carefully excised and fixed in a 3.7% formaldehyde solution for two days and subsequently embedded in paraffin for histological analyses. Serial sections of tissue specimens of 6 µm thickness were prepared and mounted on microscope slides.

### 4.5. Picrosirius Red Staining

Slices were heated to 60 °C in a heating chamber for 30 min. After rehydrating the samples, Picrosirius Red (PSR) staining was performed using the Picro Sirius Red Stain Kit (ab150681, Abcam, Cambridge, UK) according to the manufacturer’s instructions. In brief, the samples were completely covered with PSR solution in a humid chamber for 60 min, rinsed twice in acetic acid, and finally dehydrated using ethanol.

### 4.6. Collagen Quantification

PSR-stained sections were imaged by a Leica microscope (Leica DM 4000B, Leica Microsystems, Wetzlar, HE, Germany) equipped with a polarized filter (Leica ICT/Pol, Leica Microsystems, Wetzlar, HE, Germany; Figure 1B). Congruent brightfield and polarized images of aortic sections were converted to grayscales by MatLab R2022a, and media, as well as adventitia, were automatically defined as previously described [29]. The area of media or adventitia area was calculated, and the white pixels in the binarized, polarized image were counted to obtain the relative collagen content within these anatomic areas.

### 4.7. Statistical Analysis

Data analysis was performed with Student’s *t*-test for comparison of two groups and Spearman’s correlation fitting a simple linear regression curve for parameter correlation using GraphPad Prism 8.4.0 (GraphPad Software, San Diego, CA, USA). Effect size (d) and Spearman’s R (R) were calculated. Data were normally distributed as confirmed by Shapiro–Wilks- and Kolmogorov–Smirnov-test and are presented as mean ± SEM. *P*-values < 0.05 were considered statistically significant.

## 5. Conclusions

HR vascular ultrasound allows an accurate determination of changes in arterial wall morphology, but also elastic properties and arterial stiffness in mouse models of different vascular pathologies. We demonstrated thickening of the IMT, increased stiffness of the arterial wall combined with accelerated PWV, and reduced GRS in an *ApoE*^−/−^-based atherosclerosis model and in diseased aortic segments of AAA mice. These results demonstrated HR vascular ultrasound to reliably assess functional parameters even in the early stages of vascular pathologies and were supported by automated collagen quantification of the arterial wall, indicating media thickening with increased collagen deposition as a structural correlation of our physiological findings.

## Data Availability

The data generated and analyzed in this study are included in this article or may be obtained by the corresponding authors on reasonable request.

## Supplementary Materials

The following supporting information can be downloaded at: https://www.mdpi.com/article/10.3390/ijms242015261/s1.
