# Supplementary material for: Vascular Ultrasound for In Vivo Assessment of Arterial Pathologies in a Murine Model of Atherosclerosis and Aortic Aneurysm
Source: Int J Mol Sci. 2023 Oct 17;24(20):15261. doi: 10.3390/ijms242015261 (PMC10607567; doi:10.3390/ijms242015261)
Supplement: Supplementary file 1 [file ijms-24-15261-s001.zip › ijms-2644350-supplementary.pdf]

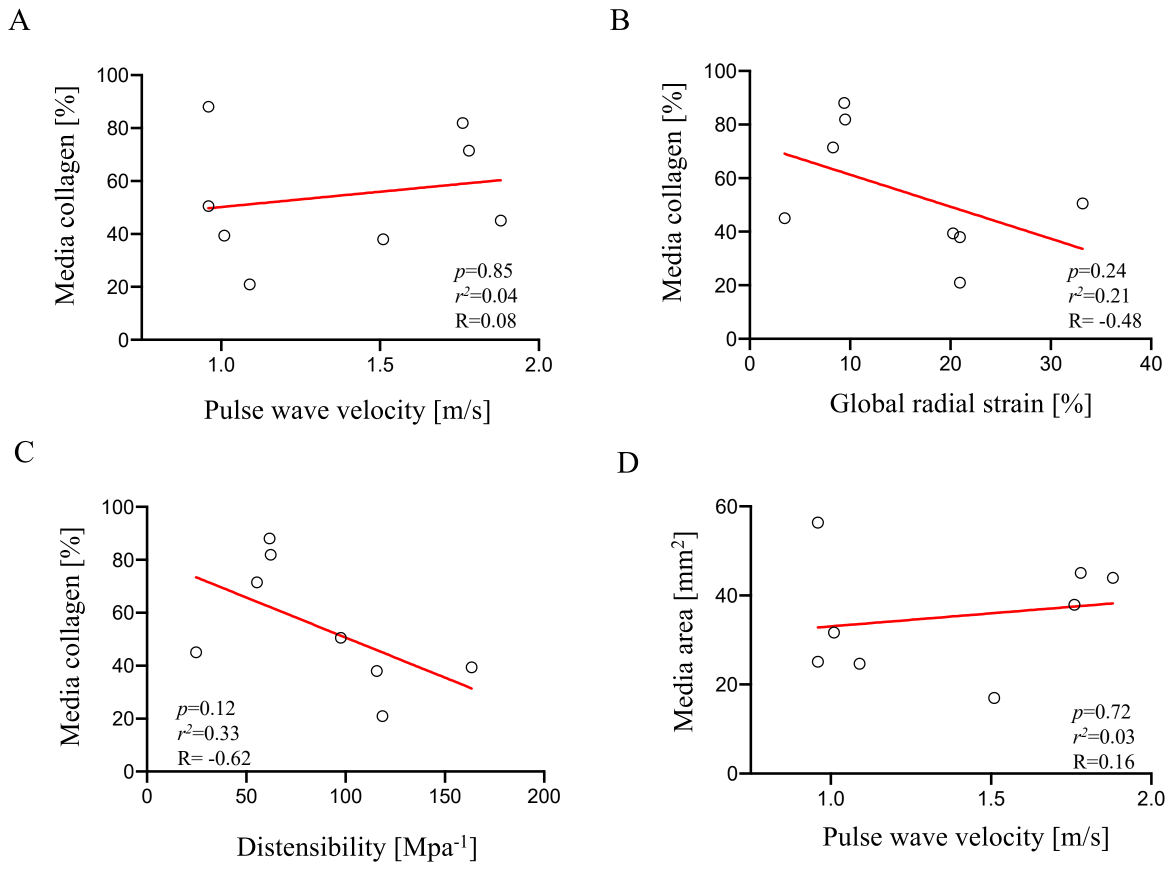

Suppl. Figure S1: Simple regression analysis of sonographic and histological parameters in abdominal aortic aneurysm. Correlation of media collagen content with global radial strain (A), distensibility (B) and pulse wave velocity (C) and of media area with pulse wave velocity (D) as measured by high resolution ultrasound. *n*=8.

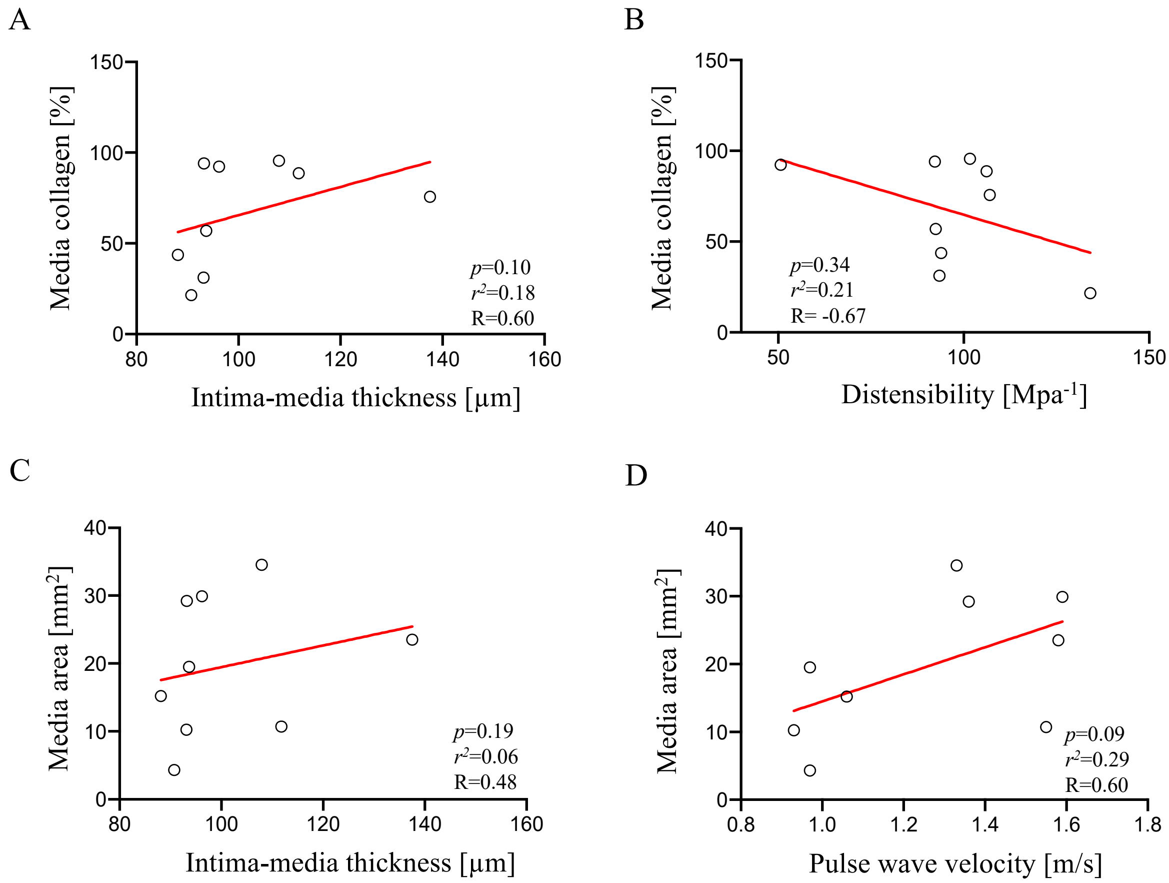

Suppl. Figure S2: Simple regression analysis of sonographic and histological parameters in atherosclerosis. Correlation of media collagen content with intima-media thickness (A) and distensibility (B) and of media area with intima-media thickness (C) and pulse wave velocity (D) as measured by high resolution ultrasound. *n*=9.
